# A Decade of College Student Hunger: What We Know and Where We Need to Go

**DOI:** 10.3389/fpubh.2022.837724

**Published:** 2022-02-25

**Authors:** Rebecca L. Hagedorn-Hatfield, Lanae B. Hood, Adam Hege

**Affiliations:** ^1^Department of Nutrition, Health, and Human Performance, Meredith College, Raleigh, NC, United States; ^2^Department of Health and Exercise Science, Appalachian State University, Boone, NC, United States

**Keywords:** food insecurity, college and university students, policy, campus programs, awareness

## Abstract

The first article on college food insecurity, published in 2009, sparked conversation on the dark secret many students face while seeking a college degree; they do not have secure access to food. Over 10 years later, numerous investigators around the globe have reported on the heightened prevalence of college food insecurity, the correlates that increase risk, and the detrimental outcomes associated with not having a secure source of food. In this manuscript, we describe the decade of research devoted to college food insecurity and provide direction for research, programs, and policies moving forward. Replicable and valid data collection methods must be utilized, campus-based program evaluation implemented and disseminated, and evidence-based policies supported to achieve realistic goals of warding off hunger and food insecurity on college campuses as well as improve the lives of individuals after post-secondary education. Collectively, stakeholders on college campuses as well as off-campus advocates can be the catalyst to creating a nutritionally secure environment and it is imperative that food insecurity be prevented on college campuses to ensure college students are able to achieve degree attainment.

## Introduction

Physiological needs, including food, are the foundation of human wellbeing according to Maslow's hierarchy of needs (1). A secure source of food is needed to thrive, yet in the United States (US), consistent availability of nutritious foods fails to be the reality for millions of Americans. In fact, food insecurity (FI), defined as an “economic and social condition of limited or uncertain access to adequate food” by the US Department of Agriculture was experienced by 13.7 million (10.5%) households in the US during 2019 (2). Despite the COVID-19 global pandemic, 2020 FI estimates remained at 10.5% of the US population (3). This finding underscores the importance and benefit of the Supplemental Nutrition Assistance Program (SNAP), with roughly 5 million additional people receiving benefits in 2020 (4). However, access and utilization of SNAP are often impeded upon by poor and unsustained policy resulting in fluctuating FI rates year to year. This may be especially true for college students who were granted expanded eligibility for the SNAP program temporarily. As COVID-19 related relief packages start to wane, impacts on FI may become more present and could have implications for several years as citizens continue to rebound from challenges presented by the pandemic.

As the cost of college has continued to rise—estimated to be a 1,200% increase since the 1980s (5)—state support for students has dwindled and reliance on student loans is customary (6). Concurrently, the demographics of college students have changed with more “non-traditional,” first-generation, minority, and lower socioeconomic (SES) status students seeking a college degree (7). Federal support for students from lower SES class families is demonstrated through the Pell Grant program, which is estimated to cover <30% of the average cost of tuition (8). We, as a society, encourage students to seek a degree as a means to end the poverty cycle, yet the burden of higher education costs results in significant student loan debt (nearly a $40,000 average) for these students (9). In fact, more than half of US undergraduates rely on federal student loans (10) but the impact of this debt burden is harmful long-term for marginalized students (11, 12). The result is a confusing balance of seeking more financial assistance but a desire to avoid debt. Today, instead of the entitled persona often portrayed of college students, the reality is many college students are skipping meals or going hungry due to the inability to afford food in conjunction with all the other necessary college expenses (rent, textbooks, lab fees, tuition, etc.) and limited financial assistance.

### The Emergence of a New Food Insecure Population

Throughout the past decade, college and university students have been identified as an emerging at-risk population. In 2009, Chaparro and colleagues published the first manuscript on college FI which reported 21% of surveyed students at the University of Hawai'i at Mānoa were food insecure (13). This report exposed a population previously overlooked in FI research. The door was opened for a conversation on a dark secret at many higher education institutions—college students were, and still are, struggling to maintain food security.

Since the initial 2009 report, numerous studies have been conducted and published. Although the FI prevalence varies across campuses, consistently, rates are higher than the national US average. A recent review found an overall weighted average of 41% of students experiencing FI (14). No type of institution is immune from this issue, with evidence from every region of the US (15, 16), at public and private universities (16, 17), and both 2- and 4-year institutions, although greater rates are often reported at 2-year colleges (18). Emerging evidence from Historically Black Colleges and Universities (HBCUs) reported more exacerbated rates with nearly 73% of students experiencing some level of FI (19). Debate on college FI estimates has occurred due to variation in reported prevalence depending on the survey method used as well as some discrepancy in how college students interpret or respond to screening questions (20). However, while the current authors agree these limitations exist and should be addressed (discussed later), qualitative studies provide a deeper look into the lives of struggling students. In fact, qualitative work highlights the hidden nature of the problems as college students express not wanting to notify family members because they “do not want them to worry” or having to lie to friends that they've “already eaten” (21). Lived experiences of food-insecure college students bring realism to the problem, no matter the precise accuracy of the quantitative measurement.

FI reaches students at both the undergraduate and graduate levels (22), across academic programs (23–28), and even collegiate athletes (29, 30). Although research suggests rates increase as students' progress academically (31), the transition away from home leads to the risk of FI starting in the first year (32). In the past decade, researchers have explored student characteristics associated with higher risk of FI. Students most at-risk include those from lower-income households (7, 16), first-generation students (33, 34), non-traditionally aged (34), and have a reported disability (7, 16). Those belonging to racial/ethnic minority groups are also disproportionately impacted (35, 36). A recent study reported that Black, first-generation college students had 296% higher odds of being food insecure compared to white, first-generation students (33). Laska et al. also reported a heightened prevalence among transgender and non-binary students compared to male or female gendered students (37).

The COVID-19 pandemic added additional layers of complexity to college FI. While households around the world faced new challenges (38), college students were confronted with the loss of employment access, campus housing, dining services, and other health-related resources as campuses shut down globally (39). Although some campuses were able to pivot delivery of emergency food programs, only students who remained in close proximity to campus were able to benefit, thus it is likely many students lost access to food resources (meal plans, campus food pantries, etc.) only available to them on campus. Hagedorn et al. reported a 15% increase in FI while Soldavini et al. found that 20% of surveyed students reported worsened FI status since the start of the pandemic (40, 41). Loss of employment was a detrimental factor and has been attributed to a 124%-473% increase in the odds of being food insecure (39–41). Wolfson and Leung highlighted broad societal challenges to employment and lack of unemployment benefits or such measures as universal basic income and direct impacts on FI (42). For college and university students, this meant those needing to work were either without sorely needed income or had to work in high-risk settings where COVID-19 was most likely to spread rapidly. Further, Lederer and colleagues emphasized that college and university students' housing insecurity became an even bigger risk as some students were dependent on on-campus housing and were forced to leave campus (43).

### Coping With FI on College Campuses

College students utilize diverse coping strategies to access food when financial resources are low or depleted. Many coping strategies place students in a precarious financial position as they must go further into debt, including consumer credit card debt and personal loans to meet their basic food needs (44). Others resort to borrowing money or leveraging social relationships to obtain food (44). Some students must choose between food and other necessities including rent, utilities, medications, transportation, and textbooks even when already working one or more jobs (45). Hagedorn and colleagues found that higher money expenditure on other items such as rent places students at greater odds of being food insecure (31). Despite lack of money to buy food, the majority of students in need of emergency food assistance were unable to access SNAP benefits before the COVID-19 pandemic due to strict eligibility guidelines for those enrolled in full-time coursework.

Although some students indicate using positive coping strategies like meal planning and food coupons, the majority do not have an adequate level of food literacy, including cooking and food budgeting skills, to regularly prepare meals. In fact, two studies identified “never cooking for themselves or others” to be a significant predictor of FI (31, 46). College students often also have busy schedules with little extra time to engage in food shopping and cooking. Moreover, there are few opportunities for students to gain these skills during college as teaching practical “life skills” is emphasized in few curriculums.

Therefore, similar to other food-insecure populations, students must rely on negative coping strategies and will often sacrifice the nutritional quality of their meals in order to get enough to eat (32, 46). This leads to a diet of cheaper, processed foods and an avoidance of more costly options like fresh fruits and vegetables. A rural Appalachia study found nearly 60% of food-insecure students would sacrifice the quality of meals as a first resort though many indicated they would like to learn about healthy eating (32). Students also utilize other behaviors associated with the “hunger and obesity paradox” such as stretching or rationing food to make it last longer, eating sporadic diets, skipping meals, utilizing food banks and pantries, or turning to community events offering free meals to fill in the gaps (45, 47). Unfortunately, many of these strategies result in feelings of shame and social stigma, making some reluctant to seek assistance. Students report “struggling silently” and being “worried that someone else needs it more than me (48).”

### A Detriment to Student Success

College students seek degree attainment as a means to a better financial future, with many low-income students looking for an opportunity to break the poverty cycle. Higher education provides a path to better health and social outcomes as well. However, FI impacts the chance of obtaining a degree (49). A recent study found food-insecure college students have 42% lower odds of graduating (50). These findings were pronounced for first-generation college students, with less than half who experienced FI finishing their education (50). This decline in graduation rate may be attributed to poor academic performance, including lower ability to focus in the classroom, which is amplified among food-insecure students (16, 31). Research has shown in students of all ages that it's hard to learn when hungry (51, 52) and college students are no exception. Ultimately, FI is a hurdle many at-risk college students are not able to overcome.

FI is also a detriment to college students' health, with food-insecure students more often reporting poor health (31, 53), although the mechanism is debated (54). The dietary intake of college students is often poor (55), regardless of FI status, and research on the association between intake and FI is mixed among this population. A recent review of 16 studies on college FI found overall poorer diet quality among food-insecure students, but statistical significance was only reported in a small number of studies (56). Poor diet quality among food-insecure college students is found among those with meal plans (57), which provides complexity to the issue as meal plans may alleviate hunger but contribute to higher obesity rates and associated health consequences among food-insecure students. Ultimately, coping strategies food-insecure college students rely on, such as diminished diet quality, lead to obesogenic behaviors that have long-term implications for chronic disease development and overall health (58). With the US spending over half of healthcare dollars on diet-related diseases (59), it is imperative to prevent obesogenic patterns in young adults.

According to reports, college students suffer from poor mental health outcomes for roughly a third of the month (10 days/month) (60). Food-insecure college students are burdened with increases in poor mental health as the likelihood of having depressive or anxious symptoms is shown to be increased among food-insecure students (60, 61). FI is further associated with worse sleep quality among college students (62), which can exacerbate poor mental and physical health outcomes (63–65) and impact student success in the classroom (66–68). In the wake of the COVID-19 pandemic, DeBate and colleagues reported food-insecure students displayed higher scores for psychological distress, loneliness, and suicidal behavior (69). Student resilience is thought to help protect students from poor mental health outcomes during times of stress (70), yet food-insecure students are reported to have reduced perception of their resilience compared to food secure students (37). As student mental health has taken a hit during the pandemic, it is even more important to consider tailored interventions for the mental health of food-insecure students.

Social experiences of food-insecure college students may also contribute to a student's departure from higher education. Social integration into the campus community is vital to students' persistence (71). Unfortunately, food-insecure college students express feeling isolated, unsupported, and an overall lack of belonging (21, 72). This disengagement from campus culture is starker among lower SES and underrepresented students (50). This lack of social presence on campus may also contribute to poor health. Willis revealed the impact social status can have on the physical and mental health of food-insecure students (54). Thus, campus efforts must strive to create a caring culture for students from all walks of life to improve academic and health outcomes.

## Discussion

Champions at higher education institutions, stakeholder groups, and policymakers alike have become aware of this issue, making it a public health priority. However, despite this acknowledgment by most within the public health realm, the data is clear; FI and hunger remain an urgent issue on college campuses. The following sections discuss recommendations for improving upon research, campus programs, and policy regarding college FI.

### Campus Programs

Research finds food pantries to be the most common campus solution, although other campus-based initiatives, such as meal share programs or campus gardens, have been implemented as well (73). Technology-based food reduction efforts have also gained traction in recent years, and are popular to reduce food waste while feeding students in need (74). The success of these programs is often unknown as evaluation is inconsistent. A group of researchers found that while a majority of surveyed campuses evaluate the impact of their campus initiatives, evaluation methods vary greatly including surveys, measuring food donations, and tracking pantry visitation (73). This makes a comparison of program reach or efficacy impossible. Moving forward, standardized evaluation methods should be utilized and there is a need for published best practices on evaluating these programs.

Student awareness and normalization of these programs must be prioritized. Barriers to the usage of food assistance programs have been investigated and found that food-insecure students report social stigma, lack of knowledge about programming, and inconvenience with hours of operation, location, and transportation (75, 76). Lack of utilization of campus programs by the most vulnerable students results in continued inequities on campus which underscore the need for targeted efforts. Raising awareness of campus-based programs is required as research shows students who are aware of programs are less likely to be food insecure (16). In fact, one study on first-generation college students found those who were aware of campus food resources had 49% lower odds of being food insecure compared to unaware first-generation students (33). Further, efforts to ensure students understand the eligibility requirements for programs is vital for program use. Increased efforts to make these programs accessible and normalized as part of the campus culture are also needed to alleviate the concerns of stigma and inconvenience.

The inclusion of holistic basic needs programs on campus would be a step in the right direction. Lederer and colleagues highlighted more emphasis and prioritization of student support services (health centers, counseling centers, student affairs staff, and target population support groups), more use of technology-based interventions and support services (which means expanded internet and broadband access), faculty training and support to assist students in need, and the use of an equity lens in campus culture, practices, and policies (43). Screening for FI at health or counseling centers may be an initial step to weaving FI outreach on campus into other programs. Further, financial and life skills training may foster the skillset some students need to navigate the monetary restrictions associated with FI.

It is important to note that these programs are merely downstream solutions. While meaningful to alleviate the symptoms of FI, reliance on these mechanisms is not enough to eradicate FI at the root.

### Policy

The Build Back Better (BBB) framework includes historic steps for improving access to higher education and nutritional security (77). We outline our support for key provisions of the BBB framework while also noting other recommendations that should be considered.

As part of the Build Back Better Act passed by the House on November, 19th 2021 investment in programs and policies that provide access to affordable higher education will help students at risk for FI (78, 79). We applaud investment in HBCUs, which aforementioned show higher rates of FI. Investment in student support services will benefit underrepresented students and ensure these programs are maintained to keep our most at-risk students in school. A small, but vital, increase in the Pell Grant by $550 will provide additional support for low-income students, although broader increases have been suggested (80, 81). Questions remain about the fate of this legislation in the Senate, with current components, including social spending being debated (82). We encourage policymakers to ensure that the Build Back Better Act includes support for the most at-risk students, both financially and through on-campus supports, to ensure attainment of a college degree is an equitable opportunity.

Student loan forgiveness was another campaigning point for President Biden (83). However, as White House Press Secretary Jen Psaki stated “a smooth transition back into repayment is a high priority for the administration” (84) loan cancellation is uncertain despite the continued delay of payments restarting. The consequence of backtracking on student loan forgiveness will be felt greatest by marginalized communities, as student loan debt often exceeds earnings for many minority students (12, 85). As aforementioned, FI forces many underrepresented students out of school, and the burden of loans without an obtained degree is even greater (86). The financial plausibility and economic impact of canceling student loan debt will continue to be debated, but without action, the inequities in America will remain. In the meantime, although recent changes have streamlined the Public Service Loan Forgiveness program (87) additional modifications are needed to reduce the payment burden for new applicants as well as the introduction of more robust repayment options for those not in public service. Scholars have provided evidence-based recommendations, such as reform of the loan servicing process and increased measures of protection for consumers, which should be utilized as policymakers navigate a path forward (88).

The BBB framework also champions improving nutritional security but has room for improvements regarding college students. As students enter college, especially those from households that have experienced an increase in FI as a result of the pandemic (89), a monumental risk of FI is present. Under the Consolidated Appropriations Act of 2021, college students who are enrolled at least part-time may be temporarily eligible for SNAP (90). While this expansion is estimated to have made an additional 3 million students eligible for SNAP (91), this change is temporary and barriers to enrollment outlined by Freudenberg and colleagues, including stigma and confusion over eligibility and the enrollment process (91), still exist. Some permanent state and federal efforts are being proposed, as identified by Laska and colleagues, but few have been implemented and progress is slow (92, 93). Permanent expansion of college student eligibility for SNAP is a necessary step to alleviate FI on college campuses. Without, the outdated exemptions for college students, as described by Freudenberg and colleagues previously (91), will continue to hinder college students in need of federal assistance. We encourage higher education administration to ensure SNAP outreach activities occur on campus to help students have access to eligibility information and support through the enrollment process. Best practices for SNAP outreach can be utilized by those seeking to improve SNAP messaging on campus (94). Further, SNAP retailers should be available on campus to ensure students have access to utilize benefits. Additionally, one issue that often correlates strongly with FI is housing affordability and accessibility, a significant challenge for students who must live off-campus (16). As such, higher education officials should collaborate with local elected officials to meet the demands of both students and local citizens. As the BBB framework highlights, this is especially important in rural areas of the country (77) and of value for the first-generation, minority, or low-income students that often reside in these communities.

Under the Biden Administration, an urge to improve nutrition security for college students as well as financial wellbeing for those stifled with debt when leaving higher education is present but the current political climate leaves many roadblocks ahead, and debate on an appropriate path to progress continues. We encourage policymakers to consider maintaining key provisions of the BBB framework as means to improve equitable access to higher education and consistent basic needs securities. In addition, the BBB framework (77) calls for enhanced support systems for families (universal pre-k, child care, and expanded child tax credits) that can help students arrive on campus better prepared and with more resources to help them be successful in the transition to life on their own.

### Research

One of the main barriers in advancing research on this topic is the inconsistent measurement methods used to quantify the prevalence and severity of FI across campuses. While most studies have utilized the USDA Food Security Survey Module (FSSM) to categorize FI with non-random or convenience samples, it is unknown whether the screening questions in this tool are appropriate for or even well-understood by the college-aged population (20, 95). Furthermore, the FSSM has a strong focus on food access, disruptions in eating patterns, and anxiety or worry about running out of food which provides a narrow understanding of a complex problem. Radimer et al. (96) proposed a broader set of indicators of FI including the nutritional quality, cultural context and acceptability, psychological aspects, and social disruptions related to food. A validated tool that encompasses all of these indicators would further contextualize the problem and offer new avenues for intervention research. Additionally, further qualitative studies are needed to provide a more holistic understanding of the problem and in-depth perspective from students who are struggling with basic needs.

Currently, there is little published literature related to campus-based interventions to address FI on college campuses. As noted, campus food pantries are intended to alleviate FI, yet monitoring the impact of these programs on reducing the prevalence or severity of FI is lacking and should be prioritized moving forward. Further, qualitative inquiries with students should be conducted to ensure the pantry meets students' needs. Improvements in social determinants of health impacted by FI, such as diet quality or mental health, should also be evaluated to understand the reach of these programs. In addition, as FI has been related to lower food and financial literacy (16, 97, 98), interventions that aim to increase these skill sets are crucial. Matias and colleagues demonstrated a semester-long nutrition intervention can decrease FI by 22% (99). While this demonstrates the potential these interventions can have, it is important to note that student needs differ across campuses. Therefore, it is imperative that researchers consider using community-based participatory approaches that include student insight into the types of programs, interventions, and policies that would be most impactful in meeting their individual needs. Allowing student participation in program development fosters buy-in and allows researchers to overcome potential barriers expressed by students (74). More research is needed to better understand approaches to enhance food access and food literacy among college students.

### Continued Awareness

Despite over a decade of data bringing awareness to the issue of college FI, including stories from food-insecure students themselves, questions remain about the emphasis being placed on college students. As part of the National Institutes of Health Virtual Workshop “Food Insecurity, Neighborhood Food Environment, and Nutrition Health Disparities: State of the Science” it was stated that “of course this work is not valid” (100). While disheartening to have the issues college students face downplayed on a national stage, those who work directly with students in need continue to champion the issue. The intention of college FI efforts is not to have policymakers be “more concerned with food insecurity among college students than, say, American Indians, very low-incomes, and persons with disabilities” as Gundersen suggests in his article to disprove college FI (101). In fact, our mission is quite the opposite. We urge policymakers to improve access to federal nutrition assistance programs for all populations that face FI, including college students. Raising awareness of college FI does not lessen awareness of other populations' FI, it heightens the message that FI is an issue in one of the wealthiest nations in the world. Anti-poverty and higher education policy reforms would benefit all populations. Our advocacy will continue to call upon the higher education system to provide equitable opportunities for students from underrepresented backgrounds, ultimately preventing more at-risk populations from becoming food insecure later in life. We encourage colleagues to support college FI efforts, spread awareness and advocate for change.

## Author Contributions

RH-H conceptualized the idea for this manuscript. All authors drafted and finalized the manuscript. All authors contributed to the article and approved the submitted version.

## Funding

RH-H and LH received funding support from Meredith College's Scholarly Productivity Grant.

## Conflict of Interest

The authors declare that the research was conducted in the absence of any commercial or financial relationships that could be construed as a potential conflict of interest.

## Publisher's Note

All claims expressed in this article are solely those of the authors and do not necessarily represent those of their affiliated organizations, or those of the publisher, the editors and the reviewers. Any product that may be evaluated in this article, or claim that may be made by its manufacturer, is not guaranteed or endorsed by the publisher.
